# Prenatal Opioid Exposure Enhances Responsiveness to Future Drug Reward and Alters Sensitivity to Pain: A Review of Preclinical Models and Contributing Mechanisms

**DOI:** 10.1523/ENEURO.0393-20.2020

**Published:** 2020-12-18

**Authors:** Gregory G. Grecco, Brady K. Atwood

**Affiliations:** 1 Department of Pharmacology and Toxicology, Indiana University School of Medicine, Indianapolis, IN 46202; 2 Indiana University School of Medicine, Medical Scientist Training Program, Indianapolis, IN 46202; 3 Stark Neurosciences Research Institute, Indiana University School of Medicine, Indianapolis, IN 46202

**Keywords:** addiction, animal models, fetal opioid, gestation, pregnancy, prenatal opioid

## Abstract

The opioid crisis has resulted in an unprecedented number of neonates born with prenatal opioid exposure (POE); however, the long-term effects of POE on offspring behavior and neurodevelopment remain relatively unknown. The advantages and disadvantages of the various preclinical POE models developed over the last several decades are discussed in the context of clinical and translational relevance. Although considerable and important variability exists among preclinical models of POE, the examination of these preclinical models has revealed that opioid exposure during the prenatal period contributes to maladaptive behavioral development as offspring mature including an altered responsiveness to rewarding drugs and increased pain response. The present review summarizes key findings demonstrating the impact of POE on offspring drug self-administration (SA), drug consumption, the reinforcing properties of drugs, drug tolerance, and other reward-related behaviors such as hypersensitivity to pain. Potential underlying molecular mechanisms which may contribute to this enhanced addictive phenotype in POE offspring are further discussed with special attention given to key brain regions associated with reward including the striatum, prefrontal cortex (PFC), ventral tegmental area (VTA), hippocampus, and amygdala. Improvements in preclinical models and further areas of study are also identified which may advance the translational value of findings and help address the growing problem of POE in clinical populations.

## Significance Statement

As the number of the infants born following prenatal opioid exposure (POE) continues to increase, there is a greater need to employ preclinical models to study the potential consequences of POE and brain and behavioral development. A large body of evidence indicates POE is associated with alterations in reward-related behavior and molecular adaptations in reward neurocircuitry. This review seeks to: (1) provide an overview of the various types of preclinical models developed over the last few decades; (2) review the numerous behavioral studies examining drug-reward related behavior in offspring with POE; and (3) discuss potential contributing molecular mechanisms to this enhanced reward phenotype observed in POE offspring.

## Introduction

The current opioid crisis has contributed to a growing population of infants exposed to opioids during prenatal development. In an analysis of nearly three million hospital deliveries, women taking long acting opioids at delivery doubled from 2006 to 2015 (Duffy et al., 2018). Estimates during this time period also indicate between 14% and 29% of pregnant women filled at least one opioid prescription during pregnancy (Epstein et al., 2013; Stover and Davis, 2015; Milliren et al., 2018; Honein et al., 2019). Opioid use in pregnancy is commonly a by-product of treating opioid use disorder (OUD) in women of reproductive age. This includes the opioid agonist medication assisted treatments methadone and buprenorphine which are the recommended treatments for OUD in pregnant women (ACOG, 2017), and the use of these opioid agonist treatments is becoming a growing majority of the total opioid exposure cases (Duffy et al., 2018). Regardless of reason for taking opioids, this rise in opioid use during pregnancy has led to a substantial number of infants born opioid dependent and subsequently developing neonatal opioid withdrawal syndrome (NOWS; previously referred to as neonatal abstinence syndrome). By 2014, NOWS reportedly rose 5-fold among a large population of Medicaid covered deliveries with one infant diagnosed every 15 min on average (Winkelman et al., 2018; Honein et al., 2019). Despite this high prevalence, the long-term implications of prenatal opioid exposure (POE) on the developing fetus remain relatively unclear. The majority of clinical studies have been small, retrospective cohorts which often lack the appropriate controls for factors such as concurrent alcohol, tobacco, or drug use, prenatal care, socioeconomic status, and many other key environmental variables that can impact prenatal and postnatal development. More recent meta-analyses have attempted to correct for these limitations, but the results from these analyses remain somewhat inconsistent which limits our understanding of the ultimate effects of POE on brain and behavioral development (Baldacchino et al., 2014; Yeoh et al., 2019; Andersen et al., 2020; Nelson et al., 2020).

## Overview of Animal Models

**Figure 1.**
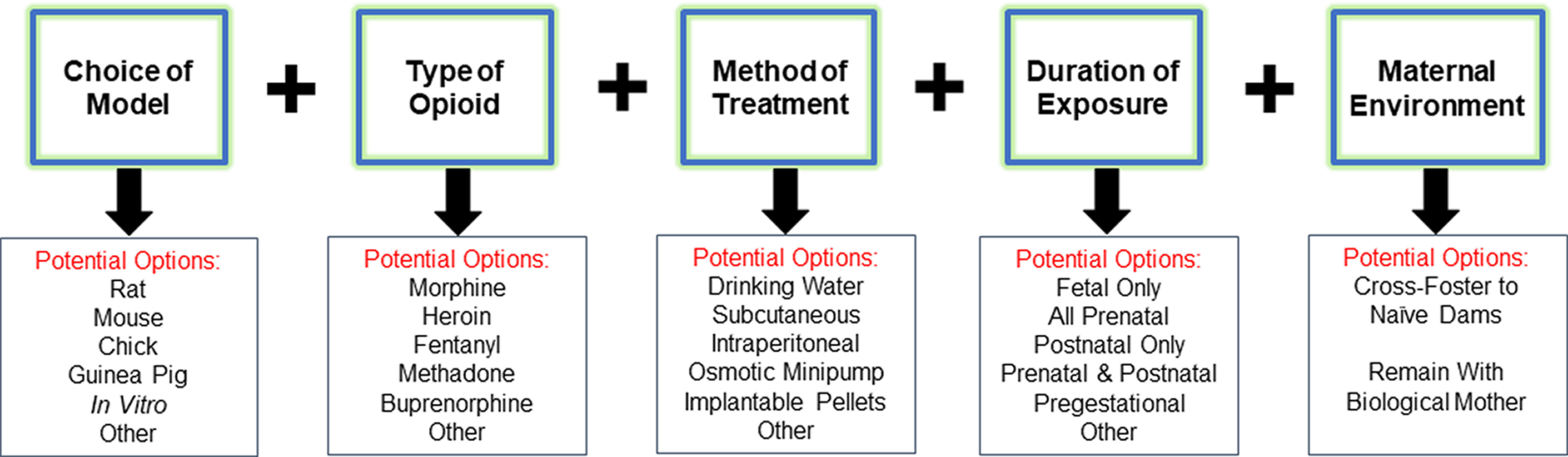
Factors varying between preclinical models of POE may differentially influence offspring outcomes. Among other factors, preclinical models of POE frequently differ between the choice of preclinical model employed, the specific type of opioid administered, the method of opioid administration, the length of prenatal exposure, and whether offspring are cross-fostered to unmanipulated, treatment naive dams at birth or remain with the biological mother. Each of these factors may differentially alter offspring outcomes which may make comparing results across studies quite difficult. See text for further details on the advantages and disadvantages of specific preclinical POE models.

For decades, researchers have pursued preclinical models of POE to begin unraveling how *in utero* opioid exposure may impact offspring development (for a recent comprehensive review of preclinical models of POE and related findings, see Byrnes and Vassoler, 2018). However, the sizeable inconsistencies between different models has complicated the generalization of preclinical findings. A summary of the factors which often differ between preclinical models of POE that may be a source of variability and that influence offspring outcomes between different studies is presented in Figure 1.

The majority of preclinical studies have used rodent models, with rats being far more common than mice, although a few groups have employed mouse models (Castellano and Ammassari-Teule, 1984; Mithbaokar et al., 2016; Kvello et al., 2019; Alipio et al., 2020; Robinson et al., 2020). Other animal models used sparingly include guinea pigs which have a placental structure more similar to humans and are born precocious (used by Olsen and colleagues; Nettleton et al., 2008; Wallisch et al., 2010) and chickens which have long been a model system for examining embryological development (Schrott and Sparber, 2004; Lucas et al., 2010; Jiang et al., 2011; Wang et al., 2017).

One of the inherent challenges to all preclinical models of POE are the differences between human and other animal prenatal development. For instance, the rodent gestational length is ∼19–22 d compared with the typical human 40-week gestational period. Rodent pups are born approximately equivalent to a second term fetus, and the rodent pup brain is thought to be most similar to a full-term neonate around postnatal days 7–8 (Clancy et al., 2007; Semple et al., 2013). Therefore, rodent studies of POE fundamentally cannot model placental opioid exposure during the third trimester of humans which is unfortunate because this is a period of continued central nervous development (Semple et al., 2013). Many researchers assume that opioids are transferred via the breastmilk to offspring at pharmacologically relevant levels allowing continued opioid exposure postnatally (Barr et al., 1998; Timár et al., 2010; Sithisarn et al., 2017; Alipio et al., 2020; Jantzie et al., 2020), but few studies are accompanied by adequate data on opioid concentrations in dams or offspring during this postnatal period. Studies which have assessed drug levels report fairly low opioid concentrations during the postnatal period (Kunko et al., 1996; Kongstorp et al., 2019; Jantzie et al., 2020). Indeed, Kongstorp et al (2019) recently reported methadone and buprenorphine levels in a rat dams and offspring on gestational day 22, postnatal day 1, and postnatal day 7. From gestational day 22 to postnatal day 1, both methadone and buprenorphine concentrations dropped over 10-fold in the brain of offspring, and plasma levels were reduced to undetectable concentrations after birth (Kongstorp et al., 2019). To circumvent the “preterm birth” issue in rodents, some researchers have chosen to directly administer opioids to rodent pups beginning at birth (Tempel et al., 1988; Tempel and Espinoza, 1992; Maeda et al., 2002; McPhie and Barr, 2009; Robinson et al., 2020), but this likely introduces significant stress to the offspring from repeated handling and painful injections. Additionally, the translational relevance of the studies that only administer opioids postnatally is questionable as it remains unlikely that many women will initiate opioid use in their third trimester of pregnancy.

The opioid of choice in preclinical studies has typical been morphine or methadone likely because of their long history of clinical usage. The growing use of buprenorphine clinically and heroin, oxycodone, and fentanyl recreationally has also translated into their usage in preclinical models (Robinson and Wallace, 2001; Chiang et al., 2010; Davis et al., 2010; Chiang et al., 2014; Devarapalli et al., 2016; Sithisarn et al., 2017; Wu et al., 2017; Griffin et al., 2019; Kvello et al., 2019; Wallin et al., 2019; Alipio et al., 2020). Less commonly used opioids, both clinically and preclinically, include the long acting methadone analogs l-α-acetylmethadol and N-desmethyl-l-α-noracetylmethadol (Schrott and Sparber, 2004; Hamilton et al., 2005). The differences between the type of opioid is likely to be consequential given the important differences in pharmacokinetics, opioid receptor efficacy and affinity, and non-opioid receptor off target binding between commonly used opioids. For instance, although buprenorphine and methadone are both appropriate medication assisted treatments for OUD during pregnancy, the pharmacological profiles are quite distinct: methadone is a full agonist at the μ-opioid receptor (MOR) and also acts as an NMDA receptor noncompetitive antagonist while buprenorphine is a partial MOR agonist with κ-opioid receptor antagonistic properties (Leander, 1987; Eap et al., 2002). Therefore, it is unlikely researchers who seek to develop preclinical models of medication assisted treatment for OUD during pregnancy will observe identical outcomes between offspring exposed to methadone and buprenorphine.

The method of opioid exposure is another aspect contributing to the inconsistencies between models. The majority of studies provide opioids to dams in a noncontingent manner either via injections (intraperitoneal or subcutaneously) or using subcutaneous osmotic minipumps or implantable pellets; the latter are often used to in an attempt to avoid the daily stress of handling and injections to mothers. However, using minipumps and pellets does not allow researchers to adjust the dosage which is an important limitation to consider as pregnancy significantly increases animal body weight and alters pharmacokinetics. Additionally, minipumps and pellets provide a steady concentration of opioids which will not reflect the peaks and troughs in drug levels normally seen in pregnant women using opioids. A translational but often less practical solution is to provide dams with free access to opioids in drinking water allowing for oral consumption throughout pregnancy (Ramsey et al., 1993; Nasiraei-Moghadam et al., 2010; Dehghani et al., 2013; Haydari et al., 2014; Alipio et al., 2020). While oral self-administration (SA) more accurately recapitulates the human condition, researchers lose the ability to control opioid exposure between experimental animals. This could be advantageous for studies seeking to correlate different levels of opioid exposure to outcomes, but problematic for studies seeking tighter control of dosing.

There is also significant variation in the duration of opioid exposure. With the exception of a handful of studies employing pregestational opioid exposure (Chiou et al., 2003; Yang et al., 2003; Lin et al., 2009; Davis et al., 2010; Mithbaokar et al., 2016; Kongstorp et al., 2019; Wallin et al., 2019), most studies initiate opioid exposure sometime during gestation (frequently gestational day 7 or greater). Although earlier opioid exposure certainly increases the length of experiments which can be burdensome, pregestational opioid exposure can impact offspring development and behavior. Opioid treatment before conception (but not gestational exposure) has been shown to alter the dopaminergic system, morphine preference, and morphine analgesia sensitivity in offspring likely through epigenetic mechanisms (Byrnes et al., 2011, 2013; Vousooghi et al., 2018; Sadat-Shirazi et al., 2019). As previously stated, this represents another area where the translational value of animal models could be improved as most women using opioids begin doing so before pregnancy.

A final area of discrepancy among preclinical models involves cross-fostering offspring at birth to an unmanipulated dam because of the concern that maternal opioid treatment or withdrawal from opioid treatment will affect care of offspring during the postnatal period (Robinson et al., 1993; Hutchings et al., 1996; Gagin et al., 1997; Robinson and Wallace, 2001; Vathy et al., 2007). Maternal opioid exposure has been demonstrated to disrupt some behavioral measures of maternal care in rodents, such as pup retrieval when removed from the nest, time spent with the litter, and active time nursing, which could impact offspring development (Slamberová et al., 2001; Wallin et al., 2019). However, it should be noted that others have not observed differences in measures of maternal care suggesting that this could be study dependent (Tan et al., 2015; Alipio et al., 2020; Kongstorp et al., 2020a). Cross-fostering does allow one to more directly examine the impact of POE on the brain and behavior without the potential confound of altered maternal care; however, cross-fostering introduces a significant early life stressor which could interact with POE to differentially alter offspring outcomes. However, in humans, rates of out-of-home care (e.g., foster care or kinship care) among children with POE reportedly range from 20% to 72% (Yeoh et al., 2019); therefore, cross-fostering in preclinical models may be an aspect of clinical relevance worth considering.

While considerable diversity exists among preclinical models of POE, many similarities have emerged regarding the effect of opioid exposure on behavioral phenotypes in adolescent and adult offspring. Numerous studies have demonstrated that POE alters the sensitivity to drug reward and alters drug reward-related behavior in offspring later in life. The remainder of the present review will focus on the impact of POE on future addiction-related behavioral phenotypes including drug SA and consumption, preference, locomotor sensitization, and tolerance. Furthermore, potential mechanistic changes in reward neurocircuitry and molecular pathways in POE offspring which may contribute to this increased vulnerability for addiction will also be examined.

## Opioid Reward

As studies using animal models of POE have grown, increasing evidence has accumulated supporting the hypothesis that POE induces long-lasting behavioral changes in offspring drug consumption, preference, sensitization, and tolerance. Understandably, most researchers have chosen to examine future sensitivity to opioid reward; although, a few studies have investigated other rewarding substances such as methamphetamine and cocaine which will be briefly reviewed in the next section. To assess this altered sensitivity, researchers have employed operant intravenous SA, oral two bottle choice or single bottle consumption, conditioned place preference (CPP), locomotor sensitization, and pain paradigms revealing tolerance to opioids. Table 1 provides a summary of the methods and findings of these opioid reward-related investigations in POE models, and select studies are discussed below.

**Table 1:** Summary of opioid addiction-related phenotypes in POE models

Animal	Opioid exposure	Characteristics of offspring studied	Findings	Reference
SA and consumption				
Wistar rats, CF	Morphine (10 mg/kg, s.c., Bid) from G7 to G9 followed by free access drinking (0.5 mg/ml in sucrose) until birth	Males at 9–10 weeks	↑ Heroin (0.063 mg/kg) and cocaine (0.125 mg/kg) administration rates with heroin active lever presses increasing significantly over sessions indicating escalation of intake	Ramsey et al. (1993)
SD Rats	Methadone (5 mg/kg, i.p., once daily) from one-week PG to P22	Males at P85–P90	↑ Oral morphine consumption (0.5 mg/ml) but not methadone (0.5 mg/ml) in 2BC	Hovious and Peters (1985)
Wistar rats	Morphine (5 up to 10 mg/kg, s.c., Bid) from G11 to G18 then gradually reduced to 0 over 4 d	Males and females at P35–P50	↑ Oral morphine consumption and preference (0.3, 0.5, and 0.7 mg/ml) using 2BC in both sexes which was reduced by maternal exercise during pregnancy	Torabi et al (2017)
Wistar rats	Morphine (0.1 up to 0.4 mg/ml in sucrose then gradually reduced after birth) free access drinking from G0 to P8	Males at P37–P49	↑ Oral morphine consumption (0.5 and 0.7 mg/ml) in 2BC compared with POE offspring born to dams that exercised during pregnancy (controls not reported)	Haydari et al. (2014)
SD rats	Morphine (0.4 mg/ml in saccharin) after mating with gradual reduction after birth	Females at P90	↑ Acquisition of morphine (0.04 mg/kg) SA behavior.	Glick et al. (1977)
SD rats, CF	Morphine (5 mg/kg up to 10 mg/kg, s.c., Bid) from G11 to G18	Males at P65–P100	↑ Morphine administration only at 1 mg/kg, but no differences at 0.3, 2, and 3 mg/kg	Riley and Vathy (2006)

CPP				
SD rats	Morphine (2 mg/kg increasing 1 mg/kg weekly, s.c., Bid) from one-week PG until birth	Males at P60	↑ Preference to morphine (1 mg/kg, i.p.) Associated with increased dopamine and serotonin turnover in the nucleus accumbens	Wu et al. (2009)
SD rats	Methadone (5 up to 7 mg/kg, s.c., Bid) from G3 to G20	Males at P64–P71	↑ Preference to methadone (4 mg/kg, s.c.) which was prevented by co-administration of dextromethorphan prenatally	Chiang et al. (2015)
SD rats, CF	Morphine (5 up to 10 mg/kg, s.c., Bid) from G11 to G18	Males at P65–P100	↑ Preference to morphine only at 0.1 mg/kg, s.c. (compared with unmanipulated controls), but no differences at 0.3, 1, 3, and 5 mg/kg	Riley and Vathy (2006)
Chicks	Morphine (1 mg/kg egg weight) on E17 and E19	P1 (sexes unspecified)	↑ Preference to morphine (1 mg/kg, i.p.)	Wang et al. (2017)
Wistar rats	Morphine (5 up to 10 mg/kg, s.c., once daily) from G0 until end of lactation	Males and females at P90–P120	↑ Preference to morphine (1 and 3 mg/kg, s.c.) in both sexes Morphine preference in offspring was more enhanced for prenatal compared with only postnatal morphine exposure	Timár et al. (2010)
Fischer rats, CF	Morphine (0.75 up to 12 mg/kg, s.c., slow emulsion solution once daily) from G12 to G18	Males and females at 10–12 weeks	↑ Preference for morphine (2 mg/kg, s.c.)	Gagin et al. (1997)
Chicks	Morphine (1 mg/kg egg weight every other day) from E5 to E8, E9 to E12, E13 to E16, or E17 to E20	P1 (sexes unspecified)	↑ Preference to morphine (1 mg/kg, i.p.) in the E9–E12 and E17–E20 exposed groups with reduced extinguishment of pairing in the E17–E20 group	He et al. (2010)

Locomotor activity and sensitization				
SD rats	Morphine (2 mg/kg increasing 1 mg/kg weekly, s.c., Bid) from one-week PG until birth	Males at P60	↑ Behavioral sensitization to morphine (1 mg/kg, i.p.) Associated with increased dopamine and serotonin turnover in the nucleus accumbens	Wu et al. (2009)
C57BL/6 mice	Morphine (10 mg/kg, s.c., Bid) from P1 to P14	Adult males and females (∼P84)	↑ Locomotor sensitization to morphine (20 mg/kg, s.c.) only mice with the AA SNP in MOR gene relative to prenatal saline exposed AA SNP mice	Robinson et al. (2020)
Chicks	Morphine (1 mg/kg egg weight) on E17 and E19	P1 (sexes unspecified)	↑ Locomotor activity during morphine CPP test (1 mg/kg, i.p.)	Wang et al. (2017)
C57BL/6J mice	Heroin (1.05 mg/kg, s.c., once daily) on G12, G15, and G18	Males and females at P28	↑ Heroin-induced acute hyperlocomotion in females which was blocked by a monoclonal antibody to 6-acetylmorphine (metabolite of heroin) administered prenatally	Kvello et al. (2019)

Tolerance and opioid-related hyperalgesia				
SD rats	Morphine (2 up to 4 mg/kg, s.c., Bid), methadone (7 mg/kg, s.c., Bid) or buprenorphine (3 mg/kg, s.c., Bid) from G3 to G20	Males at 8–12 weeks	↑ Tolerance to morphine (10 mg/kg, s.c.) in all opioid exposed groups in tail flick assay, but methadone and buprenorphine exposed offspring did not show tolerance to the respective opioid as adults	Chiang et al. (2010)
SD rats	Morphine (2 mg/kg increasing 1 mg/kg each week, s.c., Bid) from one-week PG to P30	Males and females at P14	↑ Tolerance to morphine (0.5 mg/kg, s.c.) in tail flick assay which was rescued by dextromethorphan prenatal treatment) In association with reduced NMDA receptors in the hippocampus	Tao et al. (2001)
Fischer rats, CF	Morphine (0.75 up to 12 mg/kg, s.c., slow emulsion solution once daily) from G12 to G18	Males and females at 10–12 weeks	↓ Tolerance to morphine in both sexes in tail flick and hot plate assay	Gagin et al. (1996)
SD rats	Morphine (2 mg/kg increasing 1 mg/kg each week until birth then increase 1 mg/kg every two weeks, s.c., Bid) from one-week PG to P30	Tested P14 (sexes not specified)	↑ Tolerance to morphine (2 μg/kg, i.c.v.) in tail flick assay completed under halothane anesthesia In association with reduced MOR density in the striatum, thalamus, and midbrain	Chiou et al. (2003)
CFE rats, CF	Morphine (5 up to 10 mg/kg, s.c., Bid) from G5 to G12	Males and females at 3, 5, and 11 weeks.	↑ Tolerance to chronic and acute morphine primarily in females in hot plate assays at all ages tested↑ Cross-tolerance to levorphanol in males and females	O'Callaghan and Holtzman (1976)
Wistar rats	Morphine (5 up to 10 mg/kg, s.c., once daily) from G0 until end of lactation	Males and females at P24 and P90	↑ Tolerance ED_50_ to morphine in males at P24 in tail flick assay	Timár et al. (2010)
CD1 mice	Morphine (10 up to 30 mg/kg, s.c., once daily) for 5 d PG then untreated during mating and reintroduced to morphine (10 up to 40 mg/kg, s.c., once daily) for 14 d during pregnancy	Males and females adults	↓ Tolerance to morphine (0.25 and 0.5 mg/kg) in tail flick assay	Castellano and Ammassari-Teule (1984)
SD rats	Methadone or morphine (5 mg/kg, i.p., once daily) from one-week PG to P21.	Males and females at P25 and P120	↑ Tolerance to acute methadone (2.5 mg/kg, s.c.) and morphine (3.5 mg/kg, s.c.) at P25 in both males and females with prenatal methadone exposure in tail flick assay and hot plate assay↑ Tolerance to acute morphine in P120 females in the hot plate in both opioid exposed offspring↑ Tolerance to acute methadone in P120 males in the tail flick assay in methadone exposed offspring	Hovious and Peters (1984)
SD rats	Buprenorphine (0.3 or 1.0 mg/kg, s.c., once daily) from one-week PG to P21.	Males and females at P29–P39	↑ Sensitivity to pain but no change in tolerance to morphine (5 mg/kg, s.c.) in hot plate assay	Wallin et al. (2019)
SD rats	Methadone (5 up to 7 mg/kg, s.c., Bid) from G3 to G20	Males at P30 and ∼P60	↑ Pain sensitivity but no change in tolerance to sub-chronic treatment with methadone (4 mg/kg, s.c., bid for 6 d) in the tail flick assay	Chiang et al. (2015)
SD rats	Morphine (5 up to 10 mg/kg, s.c., Bid) from G5 to G12	Males and females at P120	↑ Tolerance to the disruptive effects of morphine (4.0 and 8.0 mg/kg, i.p.) but not amphetamine in male rats during an operant responding task	Wagner et al. (1986)

Bid, bis in die (twice per day); CF, cross-fostered; CFE, Carworth Farms Elias; ED50, median effective dose; E, embryonic; G, gestational; i.c.v., intracerebroventricular; NMDAR, NMDA receptor; MOR, μ-opioid receptor; PG, pregestational; P, postnatal; SD, Sprague Dawley; SNP, single nucleotide polymorphism; 2BC, two bottle choice.

SA, often considered the “gold standard” of preclinical addiction research, is widely used in animal models for its excellent face validity to human drug consumption and the ability to quantify the strength a drug’s reinforcing effects (Sanchis-Segura and Spanagel, 2006). Both non-operant (e.g., voluntary oral consumption) and operant SA methods have been applied to the study of opioid reward in POE models, and studies have largely revealed that rodents with POE display increased opioid SA and voluntary oral opioid consumption (see Table 1). These findings support the possibility that POE enhances the preference for oral opioid solutions and increases the reinforcing effects of opioid reward relative to prenatal saline exposed controls. For instance, Ramsey et al. (1993) demonstrated that offspring with prenatal morphine exposure showed increased heroin intravenous SA rates compared with saline-exposed controls. From a non-operant perspective, both adolescent male and female rats with prenatal morphine exposure showed greater rates of oral morphine consumption and significantly enhanced preference of the morphine solution over water at all three doses examined compared with saline exposed counterparts (Torabi et al., 2017). An alternative but equally probably interpretation of this increased SA and consumption behavior may be that POE offspring are in fact less sensitive to opioid reward which motivates and drives a subsequent increase in opioid intake relative to saline-exposed controls.

CPP is another widely used behavioral assessment based in classical conditioning whereby a neutral environmental stimulus is repeatedly paired with the reinforcing properties of a drug. An increase in preference for the drug-paired environment is reflective of the positive reinforcing effects of the drug (Sanchis-Segura and Spanagel, 2006). Numerous studies in both rodents and chicks employing various CPP paradigms suggests that offspring with prenatal morphine or methadone exposure are more sensitive to the positive reinforcing properties of opioids later in life. Few studies examine how opioid exposure at different prenatal periods affect outcomes in offspring, but a noteworthy study in chicks by He et al., may suggest the period of POE plays an important role in the reinforcing properties of opioids in offspring later in life (He et al., 2010). Offspring with prenatal morphine exposure from embryonic day (E)17 to E20 exhibited increased preference for the morphine paired compartment and this drug-context preference was retained following a 72-h drug-free period; however, this increased morphine CPP and retention was not present in offspring that received morphine exposure on E5–E8, E9–E12, or E13–E16 (He et al., 2010).

Numerous drugs of abuse, including opioids, produce hyperlocomotion in rodents following treatment, and repeated administration often results in a persistent increase in motor activity known as locomotor sensitization (Wise and Bozarth, 1987; Sanchis-Segura and Spanagel, 2006). This locomotor sensitization is thought to reflect increased drug “wanting” often linked with craving and is associated with increased drug seeking and drug taking behavior (Sanchis-Segura and Spanagel, 2006). Fewer POE studies have examined locomotor activity in response to future opioid treatment; therefore, generalizations must be taken with caution. Nonetheless, enhanced locomotion has been reported in mice, rats, and chicks potentially reflecting an experience augmented drug craving in POE offspring. For, instance, male rats with prenatal morphine exposure demonstrated robust locomotor sensitization to morphine which was not present in control groups (Wu et al., 2009). Additionally, this locomotor sensitization was concurrently associated with greater morphine preference in CPP providing further evidence that this model of POE produces persistent neuroadaptations in offspring which are linked to heightened reinforcing properties of opioids (Wu et al., 2009).

Tolerance and opioid-induced hyperalgesia are common characteristics of OUD and both may contribute to an escalation of drug taking behaviors in humans (Koob, 2020). In rodent models of POE, opioid tolerance or hyperalgesia is frequently inferred from studies employing the tail flick or hot plate test which assesses the animal’s latency to respond to a noxious thermal stimulus. The greatest proportion of studies reviewed in Table 1 are devoted to examining the analgesic efficacy of opioids in animals with POE. These studies have demonstrated that many rodent models of POE have increased sensitivity to painful stimuli and higher tolerance to the analgesic properties of opioids that persists into adulthood. It remains to be determined whether the reported hyperalgesia and augmented opioid tolerance are related to other reward phenotypes such as enhanced opioid consumption or escalation of opioid intake.

## Non-Opioid Reward

Although most studies have investigated the impact of POE on opioid reward-related behaviors, others have examined the sensitivity to non-opioid rewarding substances such as cocaine and methamphetamine. Altered methamphetamine SA, CPP, and locomotor sensitization has been reported in various POE models (Chiang et al., 2014; Wong et al., 2014; Shen et al., 2016). Prenatal methadone-exposed early adolescent male rats showed heightened locomotor activity following acute and repeated methamphetamine injection and augmented persistence of this behavioral sensitization when methamphetamine-induced locomotion was examined after 4 d of abstinence (Wong et al., 2014). In contrast to this previous study, researchers reported that only adult males rats with prenatal buprenorphine exposure (and not prenatal methadone exposure) demonstrated significantly increased methamphetamine induced CPP and locomotor sensitization relative to prenatal saline controls (Chiang et al., 2014). The lack of augmented sensitization in methadone-exposed animals reported by Chiang et al. compared with Wong et al., is unclear but could be because of differences in the age of animals examined (five-week vs eight- to twelve-week-old) or the higher dose of methamphetamine used (1 vs 2 mg/kg; Chiang et al., 2014; Wong et al., 2014). Shen et al. (2016) also reported similar expression of CPP in adult males with prenatal morphine exposure compared with their saline counterparts; however, their findings revealed that morphine-exposed offspring exhibited slower extinction of CPP and a stronger methamphetamine priming-induced reinstatement of CPP behavior. Similarly, prenatal morphine-exposed animals show reduced extinction and greater methamphetamine priming-induced reinstatement in an operant methamphetamine SA task (Shen et al., 2016). These behavioral findings suggest that prenatal morphine exposure may led to greater methamphetamine seeking behavior and lowered resistance to methamphetamine relapse in adult offspring (Shen et al., 2016).

A small number of studies have also examined sensitivity to cocaine reward. In addition to greater rates of heroin SA highlighted above, Ramsey et al. (1993) also reported greater rates overall of cocaine SA in prenatal morphine exposure offspring; although, cocaine seeking did not escalate over time as heroin intake did. The model of prenatal morphine exposure by Vathy and colleagues may also show greater sensitivity to certain aspects of cocaine reward. Although, morphine-exposed offspring did not exhibit differences in cocaine SA (Vathy et al., 2007), an acute cocaine injection enhanced facilitation of intracranial self-stimulation to the medial forebrain bundle which may suggest the rewarding properties of cocaine were amplified in these animals (Vathy, 2002).

Although there is a large body of evidence to support the relationship between POE and altered reward responsiveness, a few studies stand in contrast to this theory. Because of the considerable variability in POE models, speculating what contributed to these contrary findings in these studies is challenging. Riley and Vathy (2006) is a noteworthy report that assessed morphine SA and CPP at multiple doses, but the findings largely diverge from the other SA and CPP studies in Table 1. The authors report prenatal morphine-exposed offspring demonstrated greater SA only at 1 mg/kg (not 0.3, 2, or 3 mg/kg) and greater CPP at 0.1 mg/kg (not 0.3, 1, 3, or 5 mg/kg) compared with saline-exposed counterparts. Interestingly, this model of prenatal morphine exposure characterized by Vathy and colleagues shows extensive differences in the endogenous opioid system (Rimanóczy and Vathy, 1995; Vathy, 2002; Vathy et al., 2003; Schindler et al., 2004; Slamberová et al., 2004).These findings could indicate that this model of prenatal morphine exposure is in fact more sensitive to the rewarding properties of lower doses of morphine, but the results were not consistent across all lower doses. Relative to the other studies examining SA and CPP in models of POE, the period of prenatal morphine treatment is rather narrow (7 d) which could indicate the cumulative opioid exposure during the prenatal period was not sufficient to significantly alter future behavior in a manner observed in other studies. Nonetheless, a complete explanation for the lack of apparent enhanced sensitivity to the rewarding and reinforcing properties of opioids in their prenatal morphine exposure model remains to be determined.

The findings from these reports suggest that this enhanced addictive phenotype in POE models may not be drug-specific. In conjunction with the opioid reward-related behavioral studies, the studies with cocaine and methamphetamine suggest that POE may induce persistent structural and/or functional changes in brain reward pathways during neurodevelopment that predisposes POE offspring to an enhanced addictive phenotype later in life, regardless of the rewarding substance to which access is later provided. Exactly what these mechanistic changes are and how they contribute to the behavioral phenotypes reported in POE models remains to be fully elucidated; however, the following section will review some potential contributing mechanisms to the enhanced addictive phenotypes seen in POE models.

## Potential Molecular Mechanisms Underlying Altered Reward Behavior

**Figure 2.**
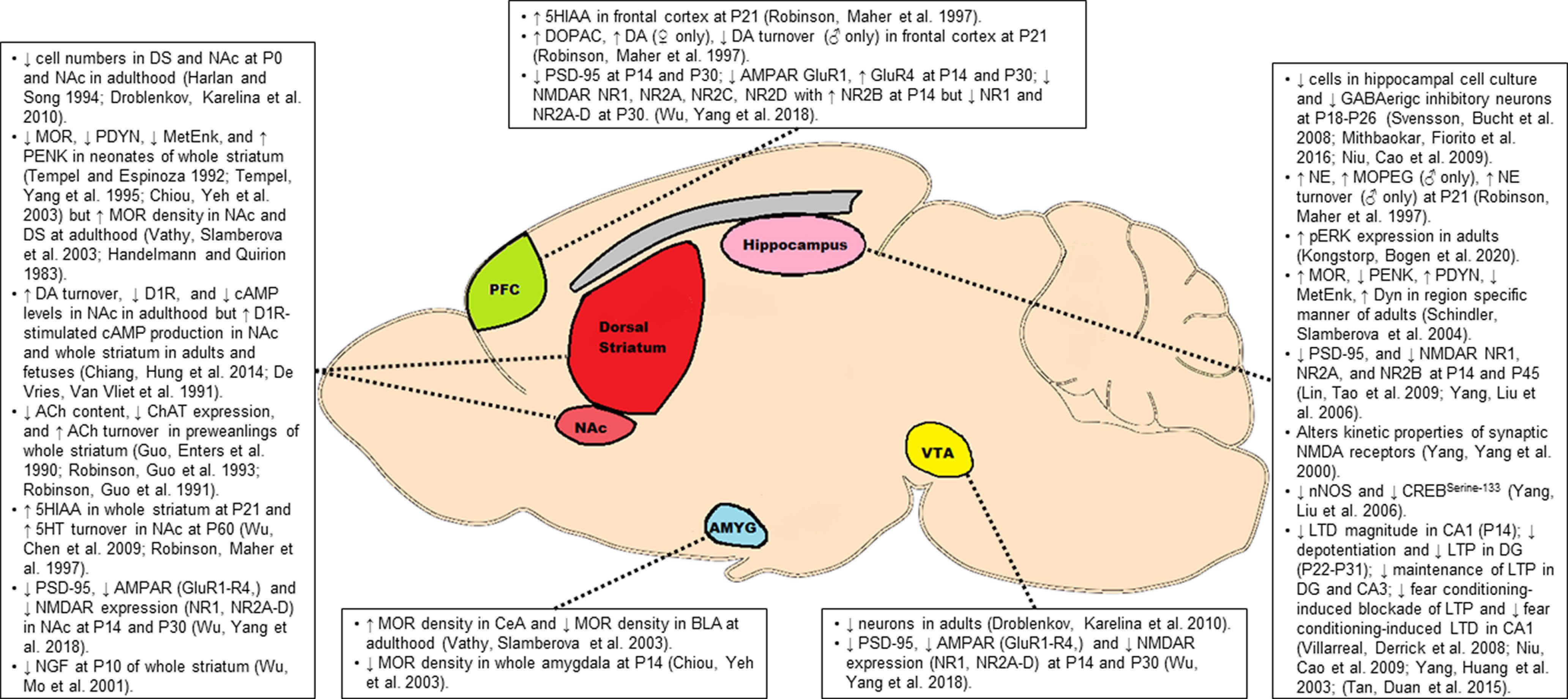
Molecular alterations in the key brain regions associated with reward. Opioids freely cross the placenta allowing access to the fetal central nervous system where they may reduce neuronal numbers and induce persistent disruptions in opioidergic, catecholaminergic, glutamatergic, and cholinergic signaling among other biochemical alterations. These molecular alterations in key nodes of the mesocorticolimbic pathway may contribute to the enhanced addictive phenotype observed in offspring with POE. See text for a more detailed description of some of these molecular alterations. 5HT, 5-hydroxytryptamine (serotonin); 5HIAA, 5-hydroxyindoleacetic acid (serotonin metabolite); Ach, acetylcholine; AMPAR, AMPA receptor; Amyg, amygdala; BLA, basolateral amygdala; CeA, central amygdala; cAMP, cyclic adenosinemonophosphate; ChAT, choline acetyltransferase; CREB, cAMP response element-binding protein; DG, dentate gyrus; DA, dopamine; D1R, dopamine receptor D1; DOPAC, 3,4-dihydroxyphenylacetic acid (dopamine metabolite); DS, dorsal striatum; Dyn, dynorphin; LTD, long-term depression; LTP, long-term potentiation; MetEnk, met-enkephalin; MOR, μ-opioid receptor; NE, norepinephrine; MOPEG, 3-methoxy-4-hydroxyphenylglycol (NE metabolite); NAc, nucleus accumbens; NGF, nerve growth factor; NMDAR, NMDA receptor; nNOS, neuronal nitric oxide synthase; PDYN, prodynorphin; PENK, proenkephalin; pERK, phosphorylated ERK; PFC, prefrontal cortex; P, postnatal day; PSD-95, postsynaptic density protein 95; VTA, ventral tegmental area.

Opioids freely cross the placental barrier entering fetal circulation and eventually the central nervous system where they presumably have direct effects on fetal brain development. A number of studies have described persistent changes in the endogenous opioid, catecholamine, glutamate, and acetylcholine neurotransmitter systems as a result of POE. Often, these molecular differences are isolated to brain regions canonically associated with addiction and reward-related behavior including the ventral tegmental area (VTA), striatum including the ventral striatal nucleus accumbens (NAc) and dorsal striatum (DS), prefrontal cortex (PFC), and limbic structures such as the amygdala and hippocampus. Despite the significant differences in molecular targets and behavioral effects, the effects of nearly all rewarding drugs eventually converge onto this mesocorticolimbic circuitry (Nestler, 2005; Lüscher and Malenka, 2011; Sulzer, 2011). Among these brain regions, the VTA-NAc dopaminergic pathway has received the greatest attention as this circuit mediates the processing of reward-related stimuli via increased dopamine release from VTA neurons in regions of the NAc (Volkow Nora and Morales, 2015). Indeed, drugs of abuse including opioids ultimately increase dopamine release via this pathway (Nestler, 2005; Sulzer, 2011), Regions of the PFC, amygdala, and hippocampus modulate this VTA-NAC pathway via glutamatergic projection which provide excitatory control over dopamine signaling (Koob and Volkow, 2016). Furthermore, these amygdalar and hippocampal projections serve to associate reward with specific emotional states and contextual environments, respectively, while PFC projections may mediate aspects of reward salience (Volkow Nora and Morales, 2015; Koob and Volkow, 2016). While decades of research have focused on this VTA-NAc pathway, the DS has received more recent attention for its involvement in generating the motor behaviors which drive compulsive drug seeking and drug taking behaviors (Everitt and Robbins, 2013; Volkow Nora and Morales, 2015; Koob and Volkow, 2016). Similar to the ventral striatum, several brains areas, including portions of the PFC, amygdala, and hippocampus project to subregions of the DS which ultimately shapes motor behaviors associated with drug reward (Everitt and Robbins, 2013; Peak et al., 2019; for a further reading on structure and functioning of reward circuitry, see Nestler, 2005; Lüscher and Malenka, 2011; Sulzer, 2011; Volkow Nora and Morales, 2015; Koob and Volkow, 2016). Highlighted studies examining the effects of POE on reward neurocircuitry are reviewed in Figure 2.

There is often difficulty in distinguishing the ultimate cause of these molecular changes when studies examine preadolescent offspring (three weeks or younger) as the differences could be because of a direct effect of exogenous opioid still present in offspring at pharmacologically relevant levels, an acute biochemical response resulting from withdrawal of opioids, or a persistent effect resulting from opioid exposure during prenatal development. Therefore, particular attention is drawn to studies below which investigate molecular differences in POE offspring beyond the first three weeks of life. At times, many of the findings from the studies described below appear at odds with one another which may pose concerns over the validity and reproducibility of the findings. However, it is important to bear in mind the timing of investigation (fetal vs early postnatal vs adolescent vs adulthood) and the differences between various preclinical models (Fig. 1) which likely contribute to the occasional contradictory findings.

Neural progenitor cells of the developing brain express opioid receptors, and immature neurons and glia also transiently express endogenous opioid peptides which suggests the endogenous opioid system may play a role in early differentiation, proliferation, and maturation of neurons and glial cells (Sargeant et al., 2008; Hauser and Knapp, 2017). Therefore, exogenous opioid exposure during a critical periods of CNS development could disrupt opioidergic signaling and impair neurogenesis and brain formation. Importantly, the endogenous opioid system is present throughout the mesocorticolimbic system exhibiting modulatory control over both dopaminergic and glutamatergic activity (Trigo et al., 2010). As such, the endogenous opioid system is implicated in both the acute and chronic effects of many rewarding substances. Using ligand binding and autoradiographic experiments, many studies indicate MOR densities and binding affinities are reduced across the whole brain and in the cortex, striatum, thalamus, hypothalamus, and amygdala of fetal and neonatal rodents with POE (Wang et al., 1986; Tempel et al., 1988, 1995; Darmani et al., 1992; Tempel and Espinoza, 1992; Belcheva et al., 1998; Chiou et al., 2003); however, this may recover during the first few weeks of life suggesting this may be an acute effect of POE or withdrawal from opioids (Tempel et al., 1988; Kongstorp et al., 2020b). In fact, studies in adult rodents indicate striatal MOR densities are actually upregulated in adulthood providing further support for the possibility that downregulation of MORs is a transient effect of POE (Handelmann and Quirion, 1983; Vathy et al., 2003). Certainly, these effects are regional dependent. For instance, male rats with prenatal methadone exposure exhibit increased pain sensitivity at both P30 and P60 which was associated with reduced MOR and nociceptin receptor mRNA expression in the spinal cord at P30 suggesting persistent downregulation in MOR expression in the afferent pain signaling pathway could underlie the hyperalgesia observed in POE offspring (Chiang et al., 2015). Dysregulation of endogenous opioid peptides such as proenkephalin, met-enkephalin, prodynorphin, and dynorphin B have also been observed during the neonatal period which may contribute to alterations in nociception (Tempel and Espinoza, 1992; Tempel et al., 1995; Schindler et al., 2004; Slamberová et al., 2004).

A recent study suggests that functioning or expression of the MOR may play an important role in the augmented locomotor sensitization response reported in POE models (Robinson et al., 2020). Using a mouse model with either the AA, AG, or GG alleles of Oprm1, Robinson et al. (2020) reported prenatal morphine-exposed male offspring harboring both A alleles exhibited greater morphine locomotor sensitization compared with their prenatal saline control AA counterparts. However, prenatal morphine did not alter the locomotor response to morphine in the AG and GG mice (Robinson et al., 2020). Single nucleotide polymorphisms in Oprm1 alter the receptor affinity for endogenous opioid ligands and availability of MOR which, in turn, can affect neurotransmission in key reward pathways (Peciña et al., 2015; Zhang et al., 2015; Taqi et al., 2019). The most commonly studied SNP in *OPRM1* is the A118G SNP in exon 1 which is associated with altered drug, alcohol, and nicotine dependence in humans (Mague and Blendy, 2010). Mice homozygous for the A allele show increased MOR expression across numerous brain regions including the NAc core, NAc shell, and VTA (Wang et al., 2012). As MORs regulate dopamine release in the VTA-NAc pathway and dopamine signaling is critical for the production of locomotor sensitization (Spanagel, 1995; Vanderschuren et al., 2001), it is possible the interaction between early life morphine exposure and altered MOR expression in reward circuitry which could underlie the differences in locomotor sensitivity in the model of POE developed by Robinson et al. Along similar lines, Wachman and colleagues have completed a number of studies in humans, demonstrating this A118G SNPs in *OPRM1* and *OPRM1* promoter methylation at certain CpG site is associated with NOWS severity and immediate clinical outcomes for children with POE (for further reading, see Wachman and Farrer, 2019). However, it remains to be determined how this SNP or other genetic and epigenetic factors involved in endogenous opioid system may interact with POE to influence developmental and behavioral differences following the neonatal period in both humans and animal models. Further work is necessary to uncover what and where in the brain persistent changes in the endogenous opioid system exist (including other opioid receptor subtypes) and how those may relate to the behaviors such as opioid tolerance and opioid reward and reinforcement in POE offspring

Growing evidence supports the hypothesis that drug-induced neuroadaptations in key cortical, striatal, and limbic circuits underlie compulsive drug seeking and drug taking behavior, and many of these pathologic neuroadaptations may be a result of an imbalance in glutamate homeostasis and signaling (Kalivas, 2009; Kalivas and Volkow, 2011; Koob and Volkow, 2016). In addition to disrupting synaptic and extrasynaptic levels of glutamate, repeated drug exposure alters surface level expression of the ionotropic AMPA and NMDA glutamate receptors disrupting the excitatory tone in the mesocorticolimbic reward system (Kalivas, 2009; Kalivas and Volkow, 2011; Olive, 2016). Additionally, drug-induced changes in glutamatergic-mediated synaptic plasticity in the mesocorticolimbic system are thought to represent the molecular correlates for many addictive behaviors (van Huijstee and Mansvelder, 2015). Deficits in synaptic plasticity (Velísek et al., 2000; Yang et al., 2003; Villarreal et al., 2008; Niu et al., 2009; Tan et al., 2015), decreased expression of AMPA, NMDA receptors and other postsynaptic density proteins (Yang et al., 2006; Lin et al., 2009; Wu et al., 2018), enhanced NMDA receptor-mediated neurotransmission, and increased NMDAR receptor-mediated current decay kinetics (Yang et al., 2000) have all been reported in various rodent models of POE with many of these effects persisting into adolescences and adulthood. These deficits in plasticity and glutamatergic signaling have primarily been reported in the hippocampus where they are often associated with impairments in spatial learning and fear conditioning (Lin et al., 2009; Niu et al., 2009; Tan et al., 2015). These POE findings parallel adult human findings that support an association between chronic opioid use and long-term deficits in cognition and memory which are hypothesized to be related to impaired hippocampal functioning (Kutlu and Gould, 2016). Using a contextual fear conditioning task, Tan et al. (2015) found that while male adult rats with prenatal morphine exposure demonstrated impaired acquisition of contextual fear memory, they maintained higher rates of freezing over repeated testing when placed in the environment previously paired with shock and this freezing behavior was resistant to extinction training. These alterations in fear conditioning were associated with impaired Schaffer collateral-CA1 plasticity: while long-term potentiation (LTP) was blocked following fear condition in controls rats as previously reported, prenatal morphine exposure prohibited fear conditioning-induced blockade of LTP (Tan et al., 2015). Prenatal morphine-exposed rats also demonstrated a small LTP in response to a low frequency stimulation protocol which normally induces long-term depression (LTD) (Tan et al., 2015). Additionally, while fear conditioning normally facilitates LTD at Schaffer collateral-CA1 synapses, prenatal morphine-exposed rats that underwent fear conditioning revealed no LTD in response to a typical LTD induction protocol while saline-exposed, fear-conditioned rats exhibited robust LTD (Tan et al., 2015). While these alterations in hippocampal plasticity and glutamatergic signaling likely contribute to contextual learning tasks, it is unclear at this time if impairments in NMDA receptor-dependent plasticity or other forms of plasticity in the hippocampus contribute to the aberrant reward-related behavioral phenotypes in models of POE. One possibility is that neonatal withdrawal from opioids may augment hippocampal-dependent, drug-context associations when POE animals are re-exposed to opioids later in life leading to increased duration in drug-paired compartments which is frequently observed in CPP as discussed above; however, additional studies are necessary to validate this speculation.

Further evidence for disrupted glutamatergic signaling is provided by Tao and colleagues whose work has revealed that co-treatment of dextromethorphan, which displays NMDA receptor antagonism, alongside POE can prevent many of the molecular and behavioral phenotypes associated with POE (Tao et al., 2001; Yang et al., 2006; Wu et al., 2009; Chiang et al., 2015). Dextromethorphan co-administration with morphine prenatally prevents the morphine antinociceptive tolerance observed in prenatal morphine-exposed offspring. Additionally, dextromethorphan prenatal co-administration also prevents the reduced density of hippocampal PSD-95, *n*NOS, phosphorylated CREB, and NMDA receptors, restores the binding affinity of MK-801 to NMDA receptors, and precludes the decreased LTD magnitude in the hippocampus which all result from prenatal morphine exposure (Tao et al., 2001; Yang et al., 2006). Another study revealed that co-administration of dextromethorphan could prevent the enhanced CPP and behavioral sensitization to repeated morphine treatment (Wu et al., 2009). Wu et al., also discovered a higher turnover rate of dopamine and serotonin in the NAc associated with this enhanced CPP and behavioral sensitization. This increased turnover rate was similarly prevented by prenatal dextromethorphan co-administration suggesting pathologic glutamatergic signaling may be upstream of disrupted dopamine and serotonin turnover in the NAc of POE offspring (Wu et al., 2009). These beneficial effects of dextromethorphan suggest that the adverse effects resulting from POE may be, in part, because of NMDA receptor dysregulation or aberrant activity at this receptor which dextromethorphan is able to prevent when co-administered during the prenatal period.

Midbrain dopamine inputs to the striatum are instrumental in mediating drug reinforcement. Additional dopaminergic projects target the amygdala, hippocampus, and PFC which assist in reward-cue/context associations (Kalivas and Volkow, 2005; Volkow et al., 2017). The projections primarily target both D1 (low affinity, G_s_-coupled) and D2 (high affinity, G_i_-coupled) receptors which are both implicated in drug reinforcement and motivation (Kalivas and Volkow, 2005; Volkow et al., 2017) Although dopamine has long been the neurotransmitter associated with reward, few studies have examined dopaminergic differences in offspring with POE. As mentioned above, increased metabolism of dopamine and serotonin was observed in the NAc (but not the dorsal striatum, medial PFC, or olfactory tubercle) of adult offspring with POE which was temporally associated with increased morphine CPP and greater morphine-induced locomotor sensitization (Wu et al., 2009). This increase in dopamine turnover may suggest that POE primes the mesolimbic pathway consisting of VTA to NAc projections to respond more robustly to a future drug reward by releasing higher amounts of dopamine at VTA terminals in the NAc relative to controls. Alternatively, changes at the dopamine receptor or signaling level may also occur following POE. D1-mediated cAMP production was increased in striatal slices of fetal rats with prenatal morphine exposure (De Vries et al., 1991). Similarly, Chiang et al. (2014) reported that prenatal buprenorphine reduced basal levels of cAMP, but augmented D1 receptor-mediated cAMP production in the NAc of adult rats suggesting this D1 mechanism is a persistent effect of prenatal buprenorphine exposure. Additionally, prenatal buprenorphine exposure reduced expression of NAc D1 receptor (without affecting D2 or D3 receptors) in adult male rats, and this was associated with enhanced methamphetamine CPP and behavioral sensitization (Chiang et al., 2014). D1 receptor activation is necessary for induction of psychostimulant locomotor sensitization (Vezina, 1996; Valjent et al., 2010); therefore, these findings suggest reduced accumbens D1 expression and/or augmented downstream signaling may be a persistent molecular disruption of the dopaminergic reward system that predisposes POE offspring to exhibit augmented behavioral sensitization when re-exposed to drugs of abuse later in life.

Numerous other molecular and biochemical changes have been reported in animal models of POE which space does not permit us to examine in detail; however, we would like to briefly acknowledge these studies for the reader’s further interest. In addition to dopaminergic disruption, POE also impacts other monoaminergic systems such as serotonin and norepinephrine, but the effects are apparently dependent on the brain region examined and the sex of offspring making generalizations difficult (McGinty and Ford, 1980; Slotkin et al., 1981; Wang et al., 1986; Vathy et al., 1994, 1995; Robinson et al., 1997; Vathy et al., 2000). While less studied relative to dopamine, both serotonin (Lovinger, 1997; Nic Dhonnchadha and Cunningham, 2008) and norepinephrine (Weinshenker and Schroeder, 2007) signaling in the mesocorticolimbic pathway are implicated in addiction. Abnormal functioning of the cholinergic system in the hippocampus and striatum has also been observed (Guo et al., 1990; Robinson et al., 1991, 1993; Yaniv et al., 2004). In the striatum, cholinergic interneurons play a critical regulatory role on striatal output which could alter motor behaviors associated with compulsive drug consumption (Bradfield et al., 2013; Augustin et al., 2018). Prenatal methadone exposure reduces total acetylcholine content likely because of increased acetylcholine turnover that persists throughout the weaning period (Guo et al., 1990; Robinson et al., 1991; Robinson et al., 1996; Yaniv et al., 2004). This disruption in acetylcholine levels could be related to the reduction in striatal NGF observed in the same model of prenatal methadone exposure as NGF is required for cholinergic neuron maturation (Wu et al., 2001; Allaway and Machold, 2017). Chronic administration of opioids to adult rodents has been shown to both upregulate pro-apoptotic and downregulate anti-apoptotic proteins (Boronat et al., 2001; Tramullas et al., 2007, 2008); similarly, POE appears to reduce cell numbers in numerous brain regions possibly by upregulating apoptotic pathways in culture and in the developing rodent brain (Harlan and Song, 1994; Svensson et al., 2008; Wang and Han, 2009; Droblenkov et al., 2010; Nasiraei-Moghadam et al., 2010, 2013; Golalipour et al., 2013). Lastly, oligodendrocytes, the myelin producing glial cells of the brain, express opioid receptors which that are thought to regulate differentiation, maturation, and myelin production (Hauser and Knapp, 2017). Preclinical studies have revealed inconsistence findings. Some studies have revealed increased myelination and myelin associated proteins (Sanchez et al., 2008; Vestal-Laborde et al., 2014) while others findings indicate damage to white matter microstructure and reduced myelin associated proteins (Jantzie et al., 2020). A small imaging study of human infants with prenatal methadone exposure did reveal alterations in microstructure in the superior longitudinal fasciculus but larger studies controlling for additional variables are necessary to substantiate these inconsistent findings (Walhovd et al., 2012).

## Conclusions and Future Directions

Numerous behavioral studies support the possibility that POE alters reward-related behavior in a manner which may enhance drug-seeking and drug-taking behaviors. Although no direct mechanistic studies have been completed, POE does appear to induce persistent molecular changes in important nodes of the classical mesocorticolimbic reward pathway which may act to “prime” these circuits to respond to rewarding substances with greater intensity later in life. As the field of preclinical POE research expands, it is possible knowledge from other models of prenatal drug exposure may lend insight to researchers studying POE. Opioids are overwhelming understudied in the field of prenatal drug exposure compared with other substances (Malanga and Kosofsky, 2003). Considerable work has been done to study the effects of prenatal alcohol, cocaine, and nicotine exposure. Prenatal alcohol exposure also increases alcohol consumption and alcohol preference in rodents with alterations in the endogenous opioid system hypothesized to underlie the alcohol intake (Fabio et al., 2015; Gaztañaga et al., 2020). Epidemiological data also supports the relationship between prenatal alcohol exposure and future risk for alcohol use disorders in humans (Alati et al., 2006). Similar evidence for an enhanced cocaine and alcohol reward phenotype is also observed in animal models of prenatal cocaine exposure (Kelley et al., 1997; Malanga and Kosofsky, 2003; Malanga et al., 2008). Prenatal nicotine exposure also appears to increase rewarding effects in offspring later in life for multiple rewarding substances including nicotine, alcohol, cocaine, and a high-fat diet chow (Franke et al., 2008; Schneider et al., 2012; Chang et al., 2013). This raises the possibility that the relationship between prenatal drug exposure and future drug reward susceptibility is neither dependent on the specific prenatal drug exposed to or the rewarding drug given access to later in life. Instead, prenatal exposure to commonly misused drugs (alcohol, nicotine, cannabinoids, psychostimulants, opioids), which may each have unique acute drug effects, may prove to produce similar behavioral phenotypes by inducing common molecular and cellular adaptations in reward circuitry during early brain development. Similar hypothesis-generating insights may develop from the large literature base studying the effects of rewarding substances on the brain of mature animals. For instance, multiple drugs of abuse disrupt LTD in the striatum of mature rodents which contributes to addiction related-behaviors (Xia et al., 2006; Kasanetz et al., 2010; Nazzaro et al., 2012; DePoy et al., 2013; Atwood et al., 2014; Abburi et al., 2016; Muñoz et al., 2018, 2020). Future studies may consider investigating disrupted striatal plasticity among other common neuroadaptations in mature animals of chronic drug exposure as potential mechanisms for the enhanced reward phenotype in models of POE.

Preclinical models offer several practical and ethical advantages over clinical studies to begin to elucidate contributing mechanisms to altered development in offspring with POE. Nonetheless, there are several aspects in which preclinical models may improve and areas which are in need of further study. In the pursuit of improving preclinical animal models, researchers should seek to model more clinically relevant scenarios of POE when developing an opioid treatment strategy. Studies which initiate opioid exposure at late stages of prenatal development are likely to have limited predictive and face validity, and therefore, have less potential to improve human health. Indeed, many recent models have introduced opioids pregestationally which is commendable (Kongstorp et al., 2019, 2020a; Wallin et al., 2019). However, further work will be necessary to discern the how opioid exposure may affect gametes before conception in comparison or in addition to the effects of opioids on the developing embryo and fetus (for further reading on parental opioid exposure and transgenerational effects, see Vassoler and Wimmer, 2020). Similarly, little is known regarding the effects of opioid on maternal neuroendocrine physiology during pregnancy and how opioid use may alter neuroendocrine physiology during pregnancy which could indirectly impact offspring development. As the POE field progresses, researchers may also want to consider the interaction between other risk factors common among women who use opioids during pregnancy (such as polysubstance exposure, resource limitations, or early life stressors) and POE when developing new translational preclinical models. For instance, a recent systematic review and meta-analysis indicated that nearly 90% of women on medications for OUD (methadone or buprenorphine) also smoked tobacco products during their pregnancy which means isolating prenatal drug exposure to methadone or buprenorphine without tobacco exposure in preclinical models may represent the exception rather than the rule in the clinic (Nelson et al., 2020). These prenatal/postnatal environmental factors may represent possible modulators for the effect of POE on brain and behavioral differences later in life (for further review, see Conradt et al., 2018). As an example, one large clinical study reported that the negative impact of POE on psychomotor performance in young toddlers no longer remained significant when controlling for covariates such as birth weight, maternal care, and the quality of the infants home environment (Messinger et al., 2004). However, a recent study revealed that POE was associated with significantly reduced motor cortex volumes and surface areas which remained significant when controlling for sociodemographic factors indicating some developmental effects are specifically associated with POE regardless of environmental interactions (Hartwell et al., 2020). Nonetheless, it remains unclear at this time how environmental stressors may interact with POE to impact future drug reward related behaviors.

To improve our current understanding of the impact of POE on reward-related behavior, researchers may consider examining other behavioral characteristics associated with addiction. Few studies have examined the resistance to extinction of drug-seeking behavior, escalation of drug intake over time, aversion-resistant or punishment-resistant drug consumption and seeking, context-induced or cue-induced reinstatement of drug-seeking behavior, or if drug withdrawal is altered in mature rodents with POE. Additionally, the vast majority of POE studies phenotyping reward-related behavior have used opioids; however, alcohol, nicotine, and marijuana remain the most likely substances the growing population of opioid-exposed infants will encounter as they mature, as these three substances constitute the most commonly available and ingested addictive substances. Yet, no studies have examined the sensitivity to alcohol, nicotine, or marijuana reward in POE offspring to our knowledge. There is a need for additional mechanistic studies as our current understanding of how POE impacts the endocannabinoid system, GABAergic functioning, neuroimmune signaling, or opioid receptors aside from the MOR is rather limited. Lastly, researchers may soon consider employing modern neuroscience tools to identify neurocircuit specific changes underlying aberrant behavior observed in POE offspring.

The pregnant woman and her developing fetus represent a particularly vulnerable population uniquely affected by the opioid epidemic, yet POE remains a relatively understudied area of the opioid field. As the opioid crisis continues unabated, there is an increasing need to understand the long-term implications of POE which may contribute to intergenerational cycles of addiction. As preclinical models of POE continue to improve their translational value and the impact of POE on reward-related behavior and neurocircuitry is further characterized, the knowledge gained from these studies will identify areas of intervention to prevent consequences in offspring resulting from opioid insult during pregnancy.

## Synthesis

Reviewing Editor: Leandro Vendruscolo, The Scripps Research Institute Decisions are customarily a result of the Reviewing Editor and the peer reviewers coming together and discussing their recommendations until a consensus is reached. When revisions are invited, a fact-based synthesis statement explaining their decision and outlining what is needed to prepare a revision will be listed below. The following reviewer(s) agreed to reveal their identity: Scott Edwards, Chris Risher.

The reviewers raised minor changes that need to be addressed. I found this manuscript to be well-organized review on the scientifically and culturally relevant subject of prenatal opioid exposure. Given the national opioid crisis and increasing number of children being born addicted to drugs, the topic is extremely timely; however, it is for this reason that I found it somewhat of a missed opportunity that only about a third of the references are from the past 5 years. Also, I thought that some of the authors' decisions unnecessarily narrowed the audience for this manuscript to one who was already fairly familiar with the reward circuitry. I would have liked to see more functional insight into the regions highlighted throughout the "Potential Molecular Mechanisms" section, aside from them just being "canonically associated with addiction and reward-related behavior" (Line 261). For example, what is the function of the striatum and VTA in addiction and reward, and why would the authors predict changes in locomotor sensitivity with increased MOR expression in these areas (Lines 304-307)? Similarly, there is a laundry list of molecules shown to be altered by POE in Figure 2, yet for most of them there is no indication of how they are thought to contribute to addiction and reward.

## Author Response

Reviewer 1

1. I think given the importance of pain in opioid use disorder, and the fact that the authors covered changes in adult pain sensitivity resulting from POE, it would be worth highlighting this in the abstract, and maybe also in the title. It could draw an additional readership.

This is an excellent suggestion given the deeply interconnected relationship between pain, tolerance, and addictions. To draw greater attention to this relationship in the manuscript, we have altered the manuscript title to include "Sensitivity to Pain" and improved the abstract by referencing the impact of POE on pain sensitivity and tolerance to pain (line 8-9, 10-11).

2. There is mention of recent meta-analyses of POE that have attempted to control for confounding variables such as socioeconomic status. The authors should briefly expand on this and comment on the role of prenatal/postnatal sociological factors as moderators of the POE-adult disorder link. For example, can we largely rule out socioeconomic factors and focus efforts on preclinical biological modeling, or will animal models never completely be expected to recapitulate the adult outcomes of POE?

The interaction between certain environmental factors (e.g. maternal stressors, adverse childhood experiences, etc.) and POE is an important concept one must absolutely consider when studying POE in humans and/or modeling this POE in the laboratory. Indeed, we draw specific attention to possible moderators of POE-developmental outcomes in the previous submission (line 491-497) which preclinical researchers may wish to consider examining. We appreciate the reviewers concern on the complex interaction between environmental stressors and POE. To expand upon this topic, we have provided additional discussion and references to important studies examining moderating effects of environmental covariates on developmental outcomes in POE children (lines 497-506). Furthermore, the relationship between early life stress and POE was also the subject of a recent review (Conradt et al., 2018. Neurobiol. Stress.) which we have now included for the readers to reference if they should want further insight (line 499).

3. Some additional care needs to be taken with certain summary terms associated with the review. For example, the authors mention in a few spots that POE "enhances the sensitivity to drug reward" or "enhances reward responsiveness" and then show evidence for escalated opioid self-administration in adulthood. This could just as easily be interpreted as less sensitivity (or tolerance) to opioid reward that drives the desire for more. This distinction basically encapsulates the debate between incentive sensitization vs. opponent process theories of drug addiction.

This is a very thoughtful comment that the reviewer raises and one that we admittedly did not consider in the original submission. This comment would appear to be particularly relevant in the self-administration section. Therefore, in the self-administration paragraph where we have discussed the possibility that POE enhances reward responsiveness to opioid self-administration and consumption, we have now included the possibility that the increases in self-administration behavior may alternatively result from reduced opioid reward sensitivity which enhances the motivation for additional opioid reward (lines 170-172). Additionally, we have removed "enhanced" from several places in this submission and replaced it with "altered" (lines 8, 142, 154, 162, 166).

4. Line 200: should be "related to other reward phenotypes".

This has been corrected

5. Line 373: "D1-mediated" should be "D1 receptor-mediated".

This has been corrected

6. Line 404: should be "inconsistent findings".

This has been corrected

Reviewer 2

1) I found this manuscript to be well-organized review on the scientifically and culturally relevant subject of prenatal opioid exposure. Given the national opioid crisis and increasing number of children being born addicted to drugs, the topic is extremely timely; however, it is for this reason that I found it somewhat of a missed opportunity that only about a third of the references are from the past 5 years.

While there has been a substantial increase in clinical studies of POE more recently, to our knowledge, there has not been a parallel increase in preclinical models of POE. Therefore, we had to draw on papers published beyond the last five years in order to thoroughly examine the relationship between POE and future susceptibility to addiction. We appreciate the reviewer's interest in more recent references and the concern is quite understandable; however, we believe this critique further underscores the desperate need for greater preclinical research on POE. Additionally, this an issue we legitimately acknowledged within the original manuscript which is that POE is a relatively understudied topic, particularly in comparison to other prenatal drug exposures (see line 456-457).

2) Also, I thought that some of the authors' decisions unnecessarily narrowed the audience for this manuscript to one who was already fairly familiar with the reward circuitry. I would have liked to see more functional insight into the regions highlighted throughout the "Potential Molecular Mechanisms" section, aside from them just being "canonically associated with addiction and reward-related behavior" (Line 261). For example, what is the function of the striatum and VTA in addiction and reward, and why would the authors predict changes in locomotor sensitivity with increased MOR expression in these areas (Lines 304-307)?

We regretfully did not include necessary background on the brain regions and circuitry which provides context to many of the studies discussed in the "Potential Molecular Mechanisms Underlying Altered Reward Behavior" section of the original submission. We thank the reviewer for bringing this to our attention and have now included a paragraph at the beginning of this section which briefly introduces the reader to reward circuitry and functional insights which we believe will contextualize the molecular mechanisms discussed in this section (line 267-284). We have also added additional text to clarify the role of MORs and the VTA-NAc pathway in locomotor sensitization to support our putative hypothesis the reviewer describes (line 330-332).

3) Similarly, there is a laundry list of molecules shown to be altered by POE in Figure 2, yet for most of them there is no indication of how they are thought to contribute to addiction and reward.

For the sake of being as comprehensive as possible, we attempted to include as many studies as possible in Figure 2 to allow readers to follow up if they had a specific interest. However, in the text, we chose to focus our discussion on molecules or neurotransmitter system which had the greatest volume of supporting literature. In several instances in the revised manuscript, we have now added supplemental text to provide context for how the various molecules/neurotransmitter systems are implicated in addiction (line 300-303, 346-351, 396-400, 426-428, and 429-431
